# Proteomic Research Reveals the Stress Response and Detoxification of Yeast to Combined Inhibitors

**DOI:** 10.1371/journal.pone.0043474

**Published:** 2012-08-27

**Authors:** Ming-Zhu Ding, Xin Wang, Wei Liu, Jing-Sheng Cheng, Yang Yang, Ying-Jin Yuan

**Affiliations:** Key Laboratory of Systems Bioengineering, Ministry of Education; Department of Pharmaceutical Engineering, School of Chemical Engineering and Technology, Tianjin University, Tianjin, People’s Republic of China; Texas A&M University, United States of America

## Abstract

The tolerant mechanism of yeast to the combination of three inhibitors (furfural, phenol and acetic acid) was investigated using 2-DE combined with MALDI-TOF/TOF-MS. The stress response and detoxification related proteins (e.g., Ahp1p, Hsp26p) were expressed higher in the tolerant yeast than in the parental yeast. The expressions of most nitrogen metabolism related proteins (e.g. Gdh1p, Met1p) were higher in the parental yeast, indicating that the tolerant yeast decreases its nitrogen metabolism rate to reserve energy, and possesses high resistance to the stress of combined inhibitors. Furthermore, upon exposure to the inhibitors, the proteins related to protein folding, degradation and translation (e.g., Ssc1p, Ubp14p, Efb1p) were all significantly affected, and the oxidative stress related proteins (e.g., Ahp1p, Grx1p) were increased. Knockdown of genes related to the oxidative stress and unfolded protein response (Grx1, Gre2, Asc1) significantly decreased the tolerance of yeast to inhibitors, which further suggested that yeast responded to the inhibitors mainly by inducing unfolded protein response. This study reveals that increasing the detoxification and tolerating oxidative stress, and/or decreasing the nitrogen metabolism would be promising strategies in developing more tolerant strains to the multiple inhibitors in lignocellulose hydrolysates.

## Introduction

A wide range of inhibitory compounds including furan derivatives, weak acids, and phenolic compounds are generated during the pretreatment and hydrolysis of lignocellulosic materials [1]. These inhibitors are toxic and strongly reduce ethanol yield and productivity by affecting the performance of fermenting microorganisms. Phenolic compounds can be incorporated into the cell membrane and cause the loss of its integrity, which impairs the membrane function as selective barriers and enzyme matrices [2]. Phenolic compounds have been suggested to cause membrane swelling and thus exert a considerable inhibitory effect in the fermentation of lignocellulosic hydrolysates [3]. Furfural has been reported to inhibit the enzymes of glycolysis [e.g., alcohol dehydrogenase (ADH), pyruvate dehydrogenase (PDH) and aldehyde dehydrogenase (ALDH)], TCA cycle, as well as the levels of ATP and ADP [4], [5]. Furfural can be reduced by NADH-dependent ADH6, which has a broad specificity of substrates [6], [7]. Undissociated weak acids can diffuse across the plasma membrane and inhibit the growth of microorganisms, resulting in depletion of the ATP content and acidification of the cytoplasm [8], [9]. Acetic acid stress affects Fps1p and Hog1p mitogen-activated protein kinase [10]. In addition, acetic acid induces the apoptosis through TOR pathway in yeast [11]. It has been reported that furfural, acetic acid and phenol all cause the accumulation of reactive oxygen species and thus induce the oxidative stress in yeast cells [4], [12]–[15]. The mechanisms of inhibition acting upon yeast during fermentation of lignocellulosic hydrolysates have been studied intensively, but mainly focusing on the effect of one inhibitor (e.g. furfural or acetic acid). Nevertheless, the molecular mechanism of ethanologenic yeast in response to multiple inhibitors is still unclear, which is an obstacle for the development of recombinant microorganisms that are tolerant to multiple inhibitors via approaches from metabolic engineering and synthetic biology.

Removal of these inhibitory compounds by physical, chemical or biochemical detoxification procedures makes the production process more complex and causes a higher cost. Thus, detoxification utilizing robust and inhibitors-tolerant microorganisms is a more favorable method [16]. The development of inhibitors-tolerant ethanologenic yeast is highly desirable for bioethanol production. However, most obtained tolerant strains are only tolerant to single inhibitor, and the strains being tolerant to combined inhibitors are urgently required. The tolerant yeast with enhanced ability to survive the combination of acetic acid (5.3 g/l), furfural (1.3 g/l) and phenol (0.5 g/l) (the concentrations of which are in accordance with the composition of ligniocellulose hydrolysates [17]) has been obtained by adaptive evaluation in our previous study (unpublished data). A mechanistic understanding of the effects of individual inhibitor in lignocellulosic hydrolysates on cell physiology will enable the development of tolerant strains, achieving rapid and efficient fermentation of the hydrolysates. Proteomics has been proven to be a useful technique for systematic understanding of biological systems as a whole under various environmental perturbations. The traditional two-dimensional electrophoresis (2-DE) has proven a powerful strategy to investigate the molecular basis of microorganisms under stress conditions.

To the best of our knowledge, this is the first proteomic analysis on the combined effects of multiple inhibitors on yeast, which is potentially useful in development of inhibitors-tolerant yeast. The combinative effect of the three inhibitors (acetic acid, furfural, and phenol) on yeast cells was analyzed using 2-DE in conjunction with MALDI-TOF/TOF-MS. The different characteristics of parental and tolerant yeasts were systematically investigated, and the different metabolisms of the two strains in response to the combined inhibitors were elucidated.

## Materials and Methods

### Strains and Fermentation Conditions

The industrial yeast strain *S. cerevisiae* (specified as ‘S’, Angel Yeast Co., Ltd., China, Product No. 80000012) used as the parental strain and the inhibitors-tolerant yeast (specified as ‘N’) obtained by evolutionary adaptation from S were used in this study. The S was pre-cultivated in YPD medium (20 g/l glucose, 10 g/l yeast extract and 20 g/l peptone), incubating for 10 h at 150 rpm and 30±0.5°C as the first grade seed. The initial optical density for inoculation of S was 0.2. The second grade seed was cultivated at an initial OD_600_ of 0.5 in YPD medium (100 g/l glucose, 10 g/l yeast extract and 20 g/l peptone), incubating overnight at 150 rpm and 30±0.5°C. The tolerant yeast was pre-cultivated in the YPD medium with three inhibitors (0.5 g/L phenol, 1.3 g/L furfural and 5.3 g/L acetic acid, specified as ‘PFA’). Other conditions for N in the first and second grade seed cultivation were the same as S. The fermentations of both S and N for proteomic study were carried out in 3 L YPD medium (100 g/L glucose, 10 g/L yeast extract and 20 g/L peptone) with/without PFA at 30±0.5°C, with a stirring rate of 300 rpm in 5 L fermenters (1.5BG-4-3000, BXBIO, Shanghai, China). The inoculum concentration of yeasts for fermentation was OD_600_ = 1.0.

The yeast knockout strains used in this study (from MATalpha library) were obtained through the *Saccharomyces* Genome Deletion Project, purchasing from Open Biosystems (Huntsville, AL). These strains were BY4742 (*MAT*α *his3*Δ*1 leu2*Δ*0 lys2*Δ*0 ura3*Δ*0*) gene deletion derivatives, including deletion of asc1D (YMR116CD), gre2D (YOL151WD), and grx1D (YCL035CD). The BY4742 and these deletion strains were cultivated at an initial OD_600_ of 0.5 in 5 ml YPD medium containing G418 (200 µg/mL), incubating overnight at 150 rpm and 30±0.5°C for the following culture. After incubation on the plate, one clone of each strain was inoculated into YPD medium for activation twice. These strains were cultivated at an initial OD_600_ of 0.1 in 100 ml YPD medium containing phenol, furfural, acetic acid, which concentration was 0.65 times of PFA, incubating for detecting the growth of these strains. For positive control, BY4742 and these deletion strains were cultivated in YPD medium without inhibitors.

### Protein Extraction and 2-DE Analysis

Yeast cells were harvested during lag phase (specified as ‘S+’ and ‘S−’ for parental yeast, and ‘N+’ and ‘N−’ for tolerant yeast in the presence and absence of inhibitors, respectively) during each fermentation process. Three completely independent sets of harvests for each sample were used in this proteomic study. The extraction procedure of protein was carried out as described previously [18]. Firstly, the collected cells were washed three times with ice-cooled Milli-Q water. Afterwards, the cell pellets were suspended into a lysis buffer (7M urea, 2M thiourea, 4% w/v CHAPS, 40 mM Tris-base, 1% w/v DNase I, 0.25% w/v RNase A, 50 mM MgCl_2_, 0.5 M Tris-HCl, pH = 7.0), and then incubated on ice. Subsequently, 1 mM PMSF was added and sonicated for 30 s. After extraction, the protein concentration was determined according to the Bradford method [19].

For 2-DE, a total of 800 mg protein was resuspended in rehydration buffer to a final volume of 350 µL. The mixture was pipetted in an 18 cm ceramics strip holder in which an immobilized linear pH gradient (IPG) strip (pH 4–7) was placed. Each strip was overlaid with mineral oil, and they were all placed on an IPGphor apparatus (GE Healthcare). The isoelectric focusing (IEF) condition was: 30 V for 12 h, 500 V for 1 h, 100 V for 1 h, 8000 V for 10 h and 10000 V for 1 h. After IEF, the strips were equilibrated for 15 min in a SDS equilibration buffer (6 M urea, 30% v/v glycerol, 2% w/v SDS, 50 mM Tris-HCl buffer of pH = 8.8, trace amount of bromophenol blue) containing 1% DTT (w/v). A second equilibration step was carried out in equilibration buffer containing 4% iodoacetamide (IAA, w/v) for 15 min. Then, the proteins were separated on 12.5% SDS-PAGE gels.

Gels were stained by Coomassie Brilliant Blue R-250. The gel images were analyzed by ImageMaster 2-D Elite version 3.01 (GE Healthcare) as described previously [18]. After spot detection and background subtraction, 2-D gels were aligned, matched and the spot volumes were quantitatively determined. Protein abundance was calculated by normalizing the protein spot volume to the total volume of all the protein spots on the gel and multiplying by 100. Fold changes were calculated as the ratio of spot intensities.

### Protein Identification by MALDI-TOF/TOF-MS

The spots of differently expressed proteins were excised, and further identified using MALDI-TOF/TOF-MS (Autoflex TOF-TOF II, Bruker Daltonics, Karlsruhe, Germany) after digested with trypsin [20]. The mass spectrometer was operated at 19 kV in the reflection mode and the mass range was 600–4000 m/z. After the peptide mass fingerprint of each protein was obtained, the LIFT mode was chosen to perform the MS/MS analysis, resulting in the MS/MS spectrum of each selected peptide. Proteins were identified by MS together with MS/MS spectra by searching NCBI database (January 31, 2009), and MASCOT (http://www.matrixscience.com. Matrix Science, London, UK) was utilized as the searching engine. The peptide and fragment mass tolerance were set at ± 100 ppm and ± 0.5Da, respectively. Carboxyamidomethylation of cysteine was set as the fixed modification, and oxidation of methionine was selected as the variable modification. A matching of two or more peptides per protein was considered as a confident detection. Proteins with scores greater than 70 were considered significantly identified (p<0.05).

*Saccharomyces* Genome Database (http://www.yeastgenome.org/) was used to get the information of identified proteins. The function of these proteins was analyzed by MIPS Functional Catalogue database (http://mips.helmholtz-muenchen.de/proj/funcatDB/).KEGG (http://www.genome.jp/kegg/pathway.html) was referenced for reconstructing major metabolic pathways. The analysis of heat maps and the change trends of the identified proteins in different conditions were carried out with Expander 4.1 software [21].

**Figure 1:**
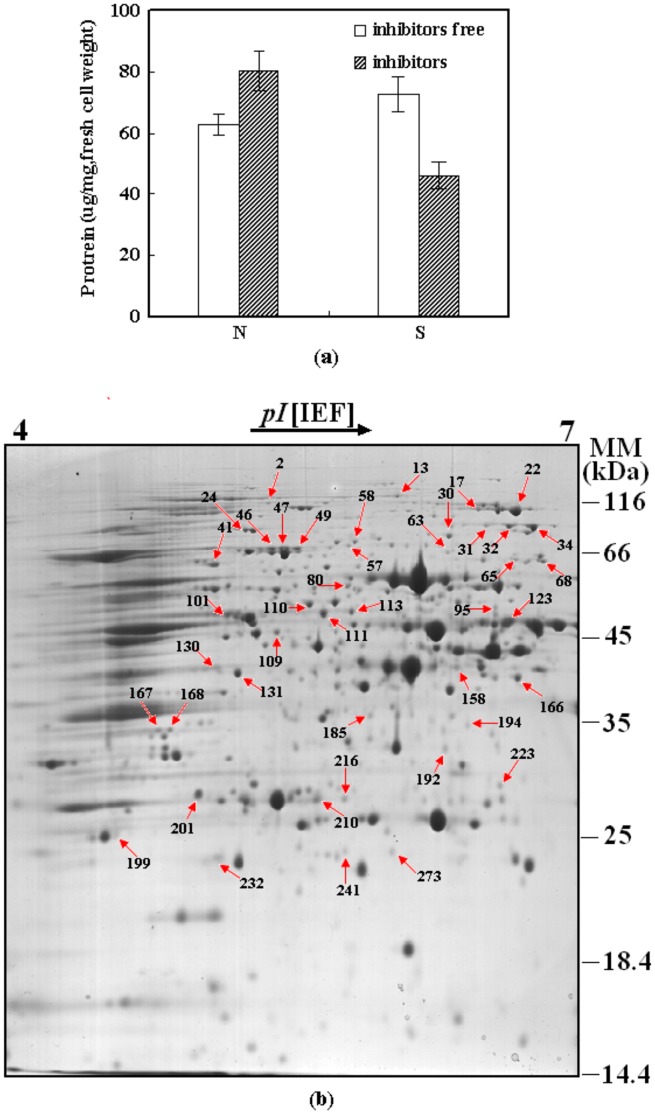
Total intracellular proteins and image of reference gel. (a) Variations of total intracellular proteins in the parental (S) and tolerant (N) yeast strains. □: in absence of PFA during fermentation; <$>\raster(85%)="rg1"<$>: in presence of PFA during fermentation; (b) Image of reference gel of parental yeast S in absence of inhibitors (S-). All the referred proteins numbered in Table 1, Table 2 and Table 3 were marked here.

**Table 1:** Differentially expressed proteins related to protein folding and stabilization, degradation, translation of S and N in the presence of PFA.

NO.	Proteins		Fold	
			S+/S−	N+/N−
	**Protein folding and stabilization**			
49	YJR045C-3	Ssc1	2.01	2.38
46	YJR045C-1	Ssc1	2.28	1.47
47	YJR045C-2	Ssc1	4.10	1.43
63	YDR258C	Hsp78	2.24	0.96
58	YOR027W-3	Sti1	2.33	1.57
41	YLR259C	Hsp60	1.76	8.41
	**Protein/peptide degradation**			
2	YBR058C	Ubp14	2.11	1.45
273	YER012W	Pre1	2.12	2.82
223	YLR178C	Dka1	2.31	2.76
216	YML092C	Pre8	2.52	2.54
30	YKL157W-2	Ape2	1.41	2.20
	**Translation**			
199	YAL003W	Efb1	0.44	0.44
201	YHR193C	Egd2	0.45	1.02
13	YLR249W-2	Yef3	2.10	0.94
167	YLR340W-1	Rpp0	2.68	2.49
168	YLR340W-2	Rpp0	4.52	2.04
192	YMR116C-2	Asc1	3.07	1.25
17	YOR133W-1	Eft1	2.50	1.08
22	YOR133W-3	Eft1	2.22	1.52

**Table 2:** Differentially expressed proteins related to amino acid and nucleotide metabolism of the parental yeast in the presence of inhibitors.

NO.	Proteins		Fold
	**AMINO ACID METABOLISM**		
	metabolism of methionine		
31	YER091C-1	Met6	2.05
32	YER091C-2	Met6	3.46
34	YER091C-3	Met6	3.47
166	YAL012W-2	Cys3	4.03
123	YLR303W-2	Met17	4.90
	biosynthesis of arginine		
113	YOL058W-2	Arg1	2.21
	biosynthesis of threonine		
80	YCR053W	Thr4	2.04
	biosynthesis of cysteine		
166	YAL012W-2	Cys3	4.03
123	YLR303W-2	Met17	4.90
	NUCLEOTIDE/NUCLEOSIDE/NUCLEOBASE METABOLISM		
24	YGL234W	Ade5,7	2.22
185	YAR015W-2	Ado1	2.34
65	YMR120C-1	Ade17	2.86
68	YMR120C-2	Ade17	3.09
130	YGR180C-1	Rnr4	2.90
57	YJR103W	Ura8	2.24

**Table 3:** Differentially expressed proteins related to amino acid and nucleotide metabolism of the tolerant yeast strain in the presence of inhibitors.

NO.	Proteins		Fold
	**AMINO ACID METABOLISM**		
	metabolism of methionine		
101	YDR502C-1	Sam2	2.55
109	YDR502C-4	Sam2	2.11
31	YER091C-1	Met6	2.04
32	YER091C-2	Met6	3.51
34	YER091C-3	Met6	3.08
95	YLR303W-1	Met17	2.51
158	YOL064C	Met22	2.21
	biosynthesis of arginine		
111	YOL058W-1	Arg1	2.17
	biosynthesis of glutamate		
110	YOR375C	Gdh1	2.77
	metabolism of polyamines		
241	YDR071C	Paa1	2.51
	others		
194	YDR272W	Glo2	3.08
30	YKL157W-2	Ape2	2.20
	NUCLEOTIDE/NUCLEOSIDE/NUCLEOBASE METABOLISM		
232	YML022W	Apt1	3.40
210	YDR399W	Hpt1	9.95
130	YGR180C-1	Rnr4	2.72
131	YGR180C-2	Rnr4	2.93
68	YMR120C-2	Ade17	2.32

## Results

### Protein Expression Analysis in Parental and Inhibitors-tolerant Yeasts

The total intracellular protein (Fig. 1a) was significantly decreased by PFA in S, while increased in N, indicating that the increase of the protein biosynthesis is an important strategy for yeast to resist the stress of PFA. However, PFA caused the degradation of proteins in parental yeast, which resulted in a decrease of total proteins. It was reported in our previous metabolomics study that the levels of most amino acids increased significantly in S, which might be due to the degradation of proteins [22]. This result validates our present finding that PFA causes protein degradation in yeast. It was analyzed by a transcriptomic study that most genes related to protein degradation were significantly up-regulated by PFA in S, whereas only a few of these genes changed in N (Fig. S1). These results further verified that PFA induced the protein degradation in S.

The 2-DE image of parental yeast S in the absence of inhibitors (S-) was shown in Fig. 1b. All the referred proteins relevant to the unfolded protein response (UPR), amino acid metabolism and nucleotide metabolism were marked in Fig. 1b, as numbered in Table 1, Table 2 and Table 3. A total of 266 protein spots were further analyzed by MALDI-TOF/TOF-MS. Taken together, the 245 spots representing 169 different proteins were successfully identified. Some differentially expressed proteins were present as multiple spots on the 2-DE gels, with one spot representing an isoform, respectively. Of these, the isoforms for part of these proteins representing same identities showed similar up- or down-regulated changes in response to PFA. The isoforms for the other proteins, representing same identities, exhibited opposite expression patterns. Likewise, similar phenomena have been observed in previous proteomics studies [23], [24], which is probably due to the posttranslational modifications. These results suggested that the isoforms of a certain protein play either the same or different roles in cells in response to PFA.

**Figure 2:**
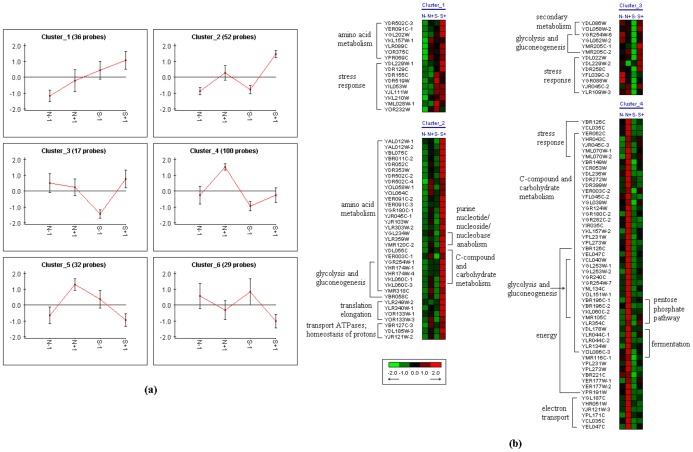
Variations of intracellular proteins. (a) Variations of the 6 clusters of proteins; (b) Heatmap of proteins with significant functions (P-value<10^−4^) in S and N.

These proteins can be categorized into six clusters according to the variations of each protein at different conditions (Fig. 2a). For the proteins that were significantly changed (P-value<10e^−4^), the variation of each protein in each cluster was showed in the heat-map (Fig. 2b). There was no protein that changed significantly (P-value<10e^−4^) in the cluster-5 and -6. Proteins in cluster-1 were expressed higher in S- than in N-, whereas the expressions of proteins in cluster-2 were similar in S- and N-. The proteins related to the amino acids metabolism existed in both cluster-1 and -2 were all decreased in the two strains upon exposure to PFA, which indicates that proteins are degraded into amino acids by PFA. On the other hand, the metabolism of amino acids may be severely affected by PFA, and lower levels of amino acids are preferential for yeast to tolerate these inhibitors. In cluster-3, proteins were up-regulated in S by PFA, while not affected significantly in N. Glycolysis related proteins in cluster-2 and -3, which were slightly affected by inhibitors in N, were of importance for the tolerance to PFA in yeast. However, these significantly increased proteins in S suggest that PFA affects the normal metabolism of glycolysis, which results in decrease in cell growth and fermentation rate. Proteins in cluster-4 exhibited an opposite trend with those in cluster-3. Proteins in N were up-regulated by PFA, while they did not change significantly in S. The proteins relevant to energy and electron transport in cluster-4 were induced more significantly in N than in S, which suggested N could produce more energy to defend against PFA. Thus, more energy are needed and produced for N to resist the stresses of PFA.

### Functional Classification of Differentially Expressed Proteins

A pair-wise comparison between parental and tolerant yeasts (N−/S-) was carried out. Moreover, yeast samples in the presence and absence of inhibitors (N+/N−, or S+/S−) were compared to investigate its response to PFA. A total of 140 protein spots represent 101 different proteins differentially expressed (i.e., changing fold higher than 2.0 or lower than 0.5) either between N− and S−, or N+ and N−, or S+ and S-. The functions of these differentially expressed proteins were analyzed by the MIPS Functional Catalogue, and the significant functions (P-value<10e^−4^) and the numbers of related protein were showed in Fig. S2. It was found that the significant functions of these differentially expressed proteins were related to carbon metabolism (e.g., C-compound and carbohydrate metabolism, amino acid metabolism, nucleotide metabolism), energy metabolism (e.g., glycolysis and gluconeogenesis), gene translation (e.g., translation elongation), cell rescue, defense and virulence (e.g., stress response and detoxification).

The proteins that are differentially expressed between S- and N- were further analyzed. We found 31 among these 52 differentially expressed proteins (P-value<10^−4^) have significant physiological functions: 6 proteins related to glycolysis and gluconeogenesis, 2 proteins related to carbon and carbohydrate metabolism, 4 proteins to amino acid metabolism, 3 proteins to nucleotide metabolism, 11 proteins to stress response, and 4 proteins to detoxification.

### Protein Expression Differences between Parental and Tolerant Yeasts

**Figure 3:**
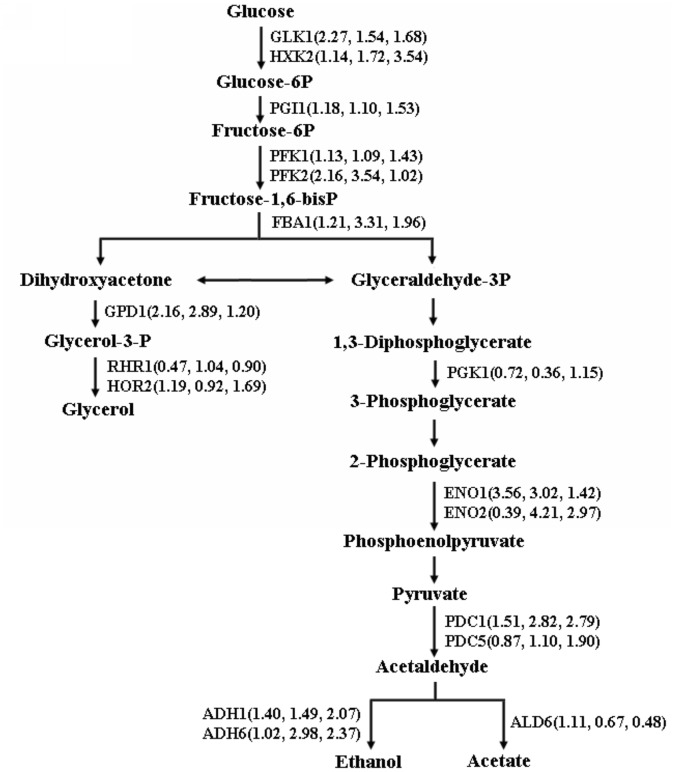
Variations of detected enzymes in glycolysis and glycerol biosynthesis. The folds in parentheses from left to right correspond to N−/S−, S+/S−, N+/N−, respectively.

**Figure 4:**
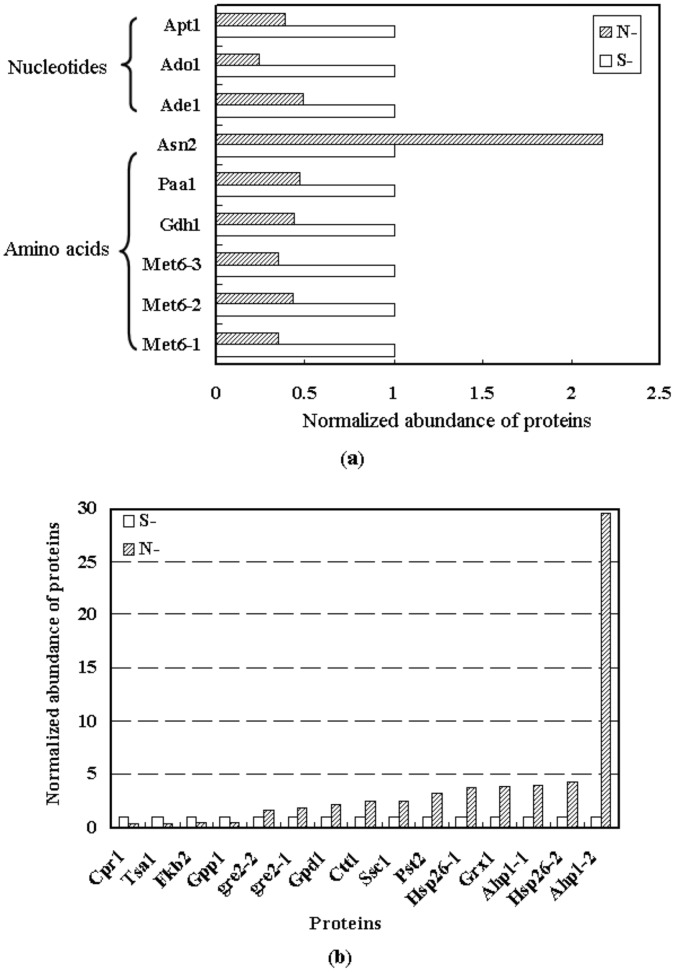
Variations of proteins related to nitrogen metabolism and stress response. (a) Differentially expressed proteins between the parental and tolerant yeast strains related to amino acid and nucleotide metabolism. (b) Normalized abundance of the proteins involved in stress response and detoxification. The abundance of proteins in S- was set as the base, i.e., 1.

Most of the important enzymes in glycolysis, such as Glk1p, Pfk2p, Gpd1p, Eno1p and Pdc1p, expressed higher in N than in S (Fig. 3), indicating tolerant yeasts possess higher metabolic activity in glycolysis which would provide higher energy to cells for better defending against PFA. Proteins related to nitrogen metabolism (including amino acid and nucleotide metabolism) were differentially expressed between S and N (Fig. 4a). Four proteins (six protein spots) were found to be related to amino acid metabolism, including two proteins in the metabolism of the aspartate family (Met6p and Asn2p). One is related to biosynthesis of glutamate (Gdh1p), and the other one related to metabolism of polyamines (Paa1p). It was shown in Fig. 4a that the expressions of most proteins related to nitrogen metabolism were lower in N than in S, which indicated that yeast decreased its nitrogen metabolism rate to reserve energy to defend against PFA.

The proteins involved in stress responses and detoxification (such as cell rescue, defense and virulence, including oxidative, osmotic, and salt stress response, unfolded protein response and oxygen and radical detoxification) were differentially expressed in the two strains. It indicates that cell rescues and defense is one of the main reasons that cause the different tolerance to PFA in yeast cell strains. Such highly expressed proteins involved in stress response and detoxification conferred N higher capability in defending against stressful conditions. The expression of these proteins was shown in Fig. 4b. It was found that most proteins involved in stress response and detoxification (e.g. Ahp1p, Hsp26p, Grx1p) exhibited higher expression in N than in S.

### Analysis of the Different Responses of Parental and Tolerant Yeasts to Combined Inhibitors

A number of proteins involved in stress response were affected by PFA in both S and N. Most of the up-regulated proteins are related with UPR, oxidative stress response, osmotic and salt stress response. The cellular responses for UPR included the enhancement of protein folding, cease of protein translation, acceleration of protein degradation. Thus, the proteins related to protein folding, degradation, and translation in both strains were analyzed in detail (Table 1), and these protein spots were marked in Fig. 1b. It is shown in Table 1 that these proteins are all significantly affected by PFA in both strains, which substantiates that UPR was induced by PFA in yeast.

**Figure 5:**
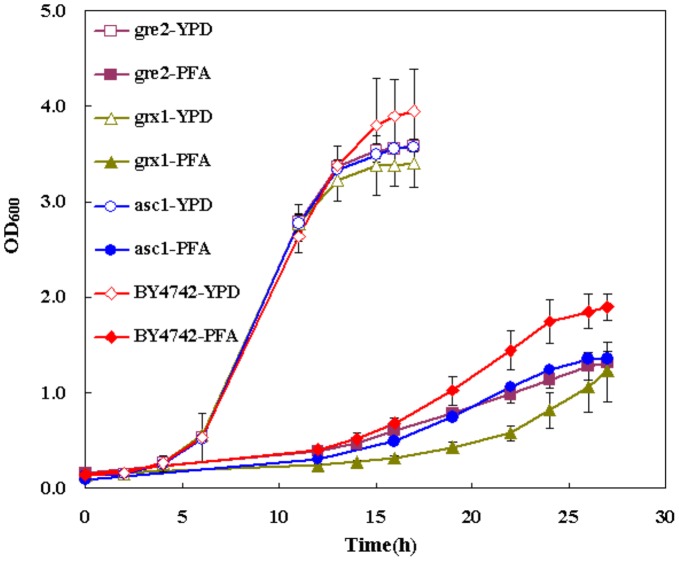
Growth of yeast with the knockdown of genes related to oxidative stress and UPR, in the absence (-YPD) and presence (-PFA) of inhibitors.

To detect whether yeast respond to PFA by evoking UPR, the yeast strains with deletion of genes in UPR (*asc1*) and oxidative stress (*grx1, gre2*) were tested for their tolerance to PFA. Interestingly, it was found that the growth of these deletion mutants was similar in the YPD medium. However, the yeast cell with knockout of UPR (asc1) and oxidative stress related genes (grx1, gre2) grew slower than the wild-type strain, and the biomass was lower in the presence of inhibitors (Fig. 5), which indicates these gene deletion strains are sensitive to the PFA, and these genes are of important for yeast to defend against PFA. In another word, the combination of PFA induces the oxidative stress and UPR in yeast.

A few important enzymes in glycolysis (e.g. Eno1p, Fba1p, and Adh1p) were up-regulated by PFA in both S and N strains (Fig. 3). However, the increase of most proteins (Fba1p, Pfk2p, Gpd1p, Eno1p, Eno2p and Adh6p) in S was higher than N, suggesting that S produced more energy derived from glycolysis to defend against PFA. Additionally, most proteins in the amino acid and nucleotide/nucleobase metabolism were up-regulated by PFA in both S and N (Tables 2 and 3). However, the affected proteins in the two strains were different, indicating that the response of the amino acid and nucleotide/nucleobase metabolism to PFA in S and N were different. Our previous metabolomics study revealed that most amino acids were significantly affected by PFA in S, while they did not have a significant alteration in N [22]. These results suggest that the amino acid metabolism is more severely affected by PFA in S. Enhancing the amino acid and nucleotide/nucleobase metabolism would be helpful for improving the tolerance of yeast to PFA.

## Discussion

**Figure 6:**
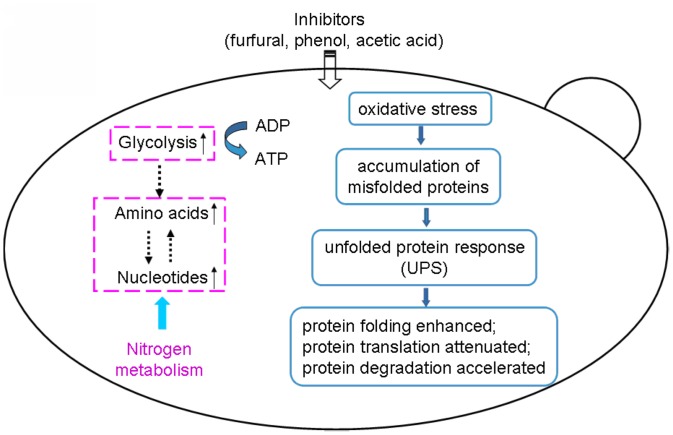
Schematic depicting the effects of PFA on the physiology of *S. cerevisiae*.

Based on our proteomic data, inhibitors-responsive proteins could be divided into two main categories. One category of proteins is related to the stress response involved in the UPR, while the other is to adjust cells’ metabolism to overcome the deleterious effects of PFA. It means that the responses of yeast to PFA have two aspects (Fig. 6). Firstly, the respond of yeast cells to the stress of inhibitors is to up-regulate the proteins in cells’ rescues and defense mechanism through UPR. Secondly, the defense mechanism of yeast cells against PFA is to regulate the carbon and nitrogen metabolism and preserve the derived energy to increase its tolerance.

It is indicated by this study that PFA causes severe oxidative stress on yeast cells, which subsequently respond to such stress by up-regulating the UPR. Many researches indicated that furfural induced the oxidative stress in yeast [4], [12], [13]. Acetic acid caused the accumulation of anions, thereby resulting in the oxidative stress [14]. Phenol induced the accumulation of reactive oxygen species [15]. It was also found in our previous study that the metabolites in response to oxidative stress (including inositol and phenylethylamine) were increased dramatically in the presence of PFA [22]. The proteins related to cell rescue and defense pathways were up-regulated to protect yeast cells from the toxic effects of PFA in the tolerant yeast strain. The UPR is an intracellular signaling pathway that is activated by the accumulation of unfolded proteins in the endoplasmic reticulum (ER) [25], [26]. The UPR not only regulates the genes related to the secretory pathway, but also the cell fate, protein metabolism, amino acid and lipid metabolism [26]. Some external factors, such as oxidative stress and treatment of chemicals, would lead to the accumulation of endoplasmic misfolded proteins. Several signaling pathways, collectively known as UPR, have evolved to detect the accumulation of misfolded proteins in the ER and activate cellular responses to maintain homeostasis and a normal flux of proteins in the ER [27]. Our present results indicate that PFA induced oxidative stress in yeast, which causes the accumulation of misfolded proteins in the UPR, including the enhancment of protein folding, arrest of the translation of most proteins, and acceleration of the degradation of proteins [27].

Yeast also defends against PFA by regulating the nitrogen metabolism. It has been found that PFA affects the normal metabolism of amino acids and nucleotides. Lower levels of amino acids and nucleotides are important for yeast to conserve energy consumption, which would benefit for the tolerance of yeast to inhibitors. Regulation of amino acids and nucleotides metabolism related enzymes would increase the tolerance of yeast. This study also indicates that yeast cells need to produce more energy and decrease the metabolism rate to reduce the energy consumption to defense against the stress of PFA. However, it may also reduce the fermentation rate which would lead to low ethanol production efficiency in the absence of PFA as concluded in our previous study [22]. It was found in the metabolomics study that the consumption rate of glucose and the production rate of ethanol were slightly lower in the tolerant yeast strain [22]. But, in the presence of PFA, the fermentation rate of the tolerant strain was much higher than the parental yeast due to its strongly enhanced tolerance to the toxicity of PFA. Amino acids are important primary metabolites, which are involved in many cellular physiological functions. It has been reported that supplementation of arginine, serine, histidine and aromatic amino acids significantly enhance the tolerance of cells to furfural [28]. Acetic acid is proved to cause the upregulation of many genes related to amino acids biosynthesis, including arginine, histidine, and tryptophan [29]. Supplementation of arginine and lysine has been proved to significantly enhance the acid tolerance of *Salmonella typhimurium* [30]. Acetic acid causes high expression level of genes encoding arginine and lysine decarboxylases [30]. Our previous study found that the genes in the amino acids metabolism (especially arginine, histidine, and tryptophan) were up-regulated by acetic acid [29]. Supplementation of methionine could increase the tolerance of yeast cells to thermal and oxidative stress, shortening the lag phase of cells [31]. These results were in accordance with our study that the PFA caused the up-regulation of proteins in the amino acids (methionine, asparagines, glutamate) metabolism. It suggests that these amino acids are of importance for the survival of yeast cells in the presence of inhibitors. In a metabolomic study, the levels of most amino acids increased significantly in the parental yeast, whereas decreased slightly in the tolerant yeast [22]. It indicates that the changes of amino acids are not only due to the regulation proteins in the amino acids biosynthesis, but also the protein degradation discussed above.

It is found that S needed to produce more energy derived from glycolysis to defend against the stress and repair the damage caused by PFA. Proteomic researches on the responses of yeast to furfural revealed that furfural induced the stress response and many aspects of metabolism were interrupted, including glucose fermentation, tricarboxylic acid cycle, and glycerol metabolism [4], [32]. Metabolic flux analysis also showed that furfural affected glycolytic and TCA fluxes, which were involved in energy metabolism [13]. Undissociated weak acids are liposoluble and can diffuse across the plasma membrane into the cytosol, which were proposed to be an important reason inhibiting the growth of microorganisms [33]. With the increase of intracellular acetic acid, more membrane ATPase is needed to pump protons out of the cell at the expense of ATP hydrolysis [9]. At high acetic acid concentrations, the proton pumping capacity of cells is exhausted, resulting in the depletion of ATP content, dissipation of the proton motive force, and acidification of cytoplasm [33]. Thus, much more energy is needed to defend against the stress of PFA, preventing the depletion of cellular energy. The increment of energy for N was significantly less than S, due to the higher tolerance of N to PFA. In the present study, most detected proteins related to energy were involved in glycolysis and gluconeogenesis. Alcohol dehydrogenases (ADH) are NADH-dependent enzymes that play a key role in the reduction of furfural to less toxic furfuryl alcohol [34]–[36]. In this study, Adh1p and Adh6p were both up-regulated by PFA in S and N, suggesting the two enzymes were crucial for the cellular detoxification of furfural. Glycerol 3-phosphate dehydrogenase (Gpd1p), the most important enzyme in the biosynthesis of glycerol, was up-regulated only in S, but not in N. It was reported that glycerol was formed to regenerate excess NADH to maintain the intracellular redox balance [37], [38]. Our results further indicate that furfural acts as an alternative redox sink that can oxidize the excessive NADH. The reduction of furfural is dependent upon ADH [3], [13], which would improve the tolerance of yeast to PFA.

In all, two main strategies are found for yeast to defense against the stress of PFA. One strategy is for yeast to have higher capability in its detoxification and toleration of oxidative stresses. The other strategy is to decrease the metabolic rate of nitrogen to reduce the consumption of energy.

## Supporting Information

Figure S1 **Differentially expressed genes related to protein degradation of parental and tolerant yeast in presence of inhibitors.** ‘+’: in presence of three inhibitors during fermentation; ‘−’: in absence of inhibitors during fermentation. 1: lag phase; 2: exponential phase.
(TIF)

Figure S2 **Significant functions (P-value<10e^−4^) in differentially expressed proteins.** The number represents the percentage of protein spots identified in each functional catalog.
(TIF)
