# Supplementary figures and images for: Proteomic Research Reveals the Stress Response and Detoxification of Yeast to Combined Inhibitors
Source: PLoS One. 2012 Aug 27;7(8):e43474. doi: 10.1371/journal.pone.0043474 (PMC3428360; doi:10.1371/journal.pone.0043474)

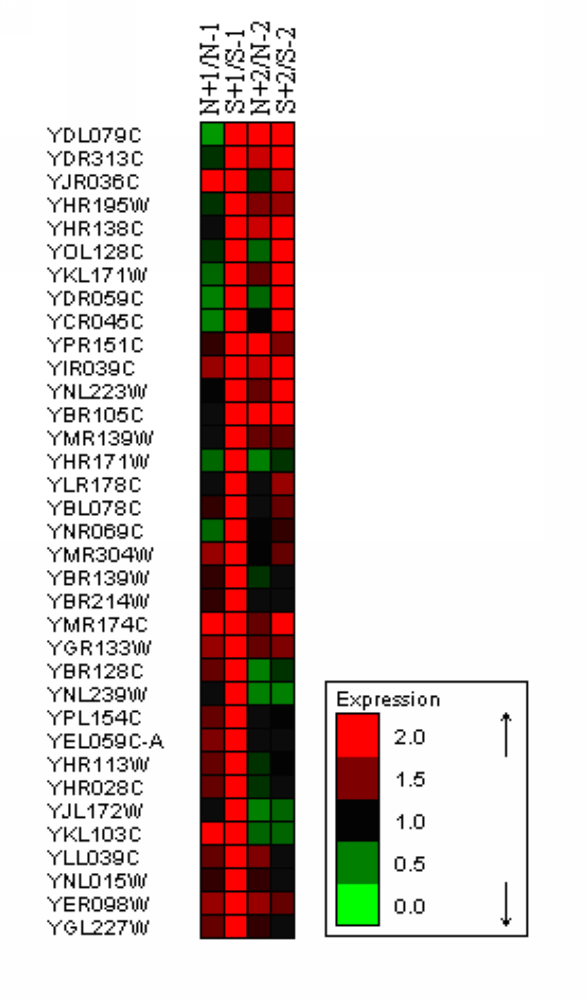

Supplement: Figure S1 — Differentially expressed genes related to protein degradation of parental and tolerant yeast in presence of inhibitors. ‘+’: in presence of three inhibitors during fermentation; ‘−’: in absence of inhibitors during fermentation. 1: lag phase; 2: exponential phase. (TIF) [file pone.0043474.s001.tif]

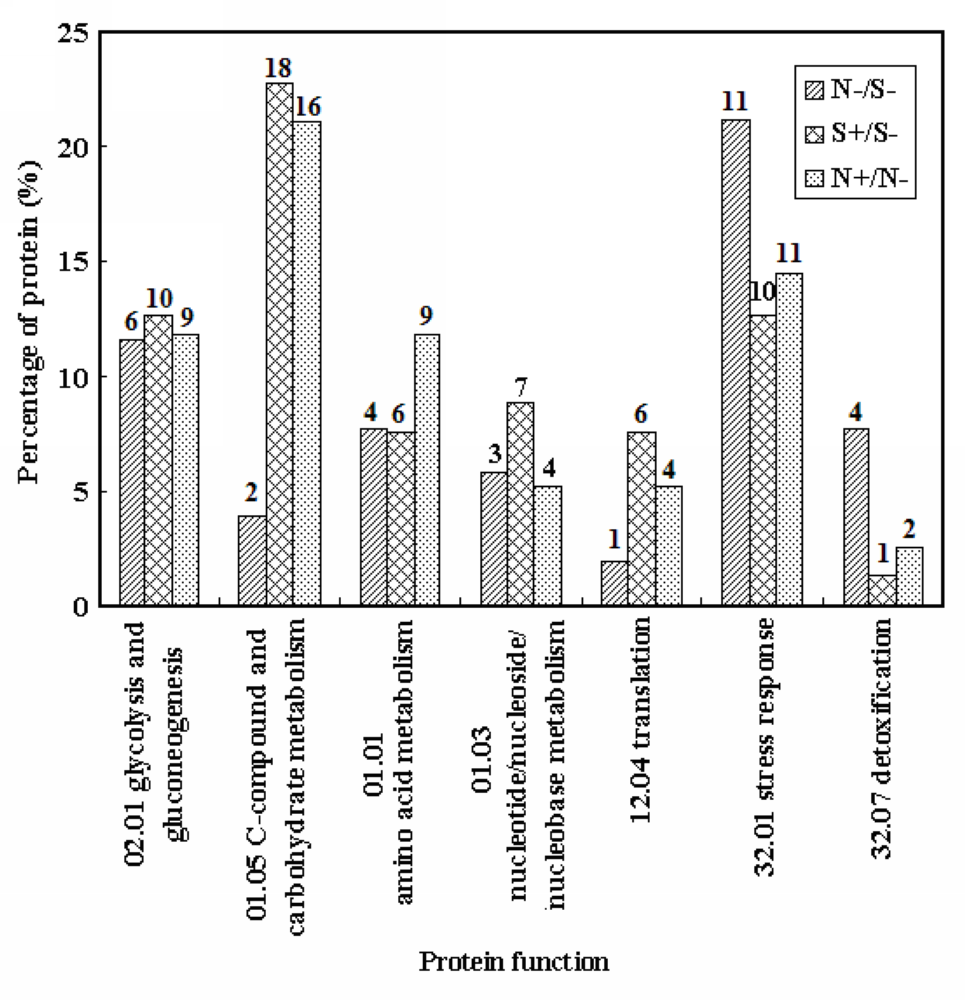

Supplement: Figure S2 — Significant functions (P-value<10e−4) in differentially expressed proteins. The number represents the percentage of protein spots identified in each functional catalog. (TIF) [file pone.0043474.s002.tif]
